# Genome Sequences of Newcastle Disease Virus Strains from Two Outbreaks in Indonesia

**DOI:** 10.1128/MRA.00205-20

**Published:** 2020-06-04

**Authors:** Phuong Thi Kim Doan, Mohamad Indro Cahyono, Mohammad Rabiei, Putri Pandarangga, Milton M. McAllister, Wai Yee Low, Rick Tearle, Indi Dharmayanti, Simson Tarigan, Risa Indriani, Jagoda Ignjatovic, Farhid Hemmatzadeh

**Affiliations:** a School of Animal and Veterinary Sciences, The University of Adelaide, Adelaide, Australia; b Faculty of Animal and Veterinary Sciences, Tay Nguyen University, Dak Lak, Viet Nam; c Department of Veterinary Pathology, Nusa Cendana University, Kupang, Indonesia; d Indonesian Research Centre for Veterinary Science, Bogor, West Java, Indonesia; e School of Veterinary Science, The University of Melbourne, Melbourne, Victoria, Australia; f Davies Research Centre, School of Animal and Veterinary Sciences, The University of Adelaide, Adelaide, Australia; Portland State University

## Abstract

The genomes of two newly emerged Newcastle disease virus strains, chicken/Indonesia/Mega/001WJ/2013 and chicken/Indonesia/Cimanglid/002WJ/2015, from disease outbreaks in chickens in Indonesia are reported. Phylogenetic analysis of different genotypes of Newcastle disease virus using the F gene coding sequences suggests that these two strains belong to genotype VII.2, in class II of avian paramyxoviruses.

## ANNOUNCEMENT

Newcastle disease (ND) is one of the most severe infectious diseases of chickens. The causative agent, ND virus (NDV), is a member of the avian genus *Orthoavulavirus* (subfamily *Avulavirinae*, family *Paramyxoviridae*) (1). NDV is a single-stranded, nonsegmented, negative-sense, and enveloped RNA virus with six major structural proteins in the order 3′-NP-P-M-F-HN-L-5′ (2, 3). NDV has been divided into two classes; class I represents avirulent strains, while class II represents virulent and nonvirulent strains (4–6). Recent ND outbreaks have appeared in commercial chickens, even vaccinated flocks, leading to mortality rates of 70 to 80%, and are caused mainly by highly virulent genotype VII NDVs (6, 7).

The two virus strains in this study were collected from two brain samples from vaccinated chickens in two different NDV outbreaks in Indonesia in 2013 and 2015. The samples were processed based on the OIE guidelines for laboratory procedures for isolating the virus (8) and then were inoculated into embryonated chicken eggs, followed by collection of allantoic fluid. RNA purification was performed using the viral RNA minikit (Qiagen, USA). cDNA libraries were prepared using random hexamers with the stranded mRNA-Seq kit (Kapa Biosystems, USA) according to the manufacturer’s instructions. The resulting cDNAs were sequenced using the Illumina MiSeq platform 600-cycle kit v3, generating 2 × 300-nucleotide reads, and the library size was checked on a Bioanalyzer 2100 using the high-sensitivity DNA kit (Agilent Technologies, Germany). Adaptors were removed using Trimmomatic v0.36 (9); 616,471 and 794,856 reads for samples 1 and 2, respectively, were *de novo* assembled using Unicycler v0.4.4 with default parameters (10) and visualized with Bandage (11). Assembled contigs from each sample were compared to the NCBI nonredundant/nucleotide collection using BLASTn (12). Two NDV contigs with a genome GC content of 46% and a length of 15,192 nucleotides were identified for the chicken/Indonesia/Mega/001WJ/2013 (Mega/001WJ) and chicken/Indonesia/Cimanglid/002WJ/2015 (Cimanglid/002WJ) strains. The Mega/001WJ and Cimanglid/002WJ contigs had average coverage of 573× and 1,837×, respectively, and 97.97% and 98% identity to the Sukorejo strain (GenBank accession number HQ697255), respectively. A gap in the sequence of Mega/001WJ was closed using reverse transcriptase PCR (Qiagen) and Sanger sequencing (13), and BioEdit v7.2 was used to merge sequences (14). Geneious release 10.1.3 and ORFfinder were used to annotate genes and to confirm open reading frames, respectively (12).

**FIG 1:**
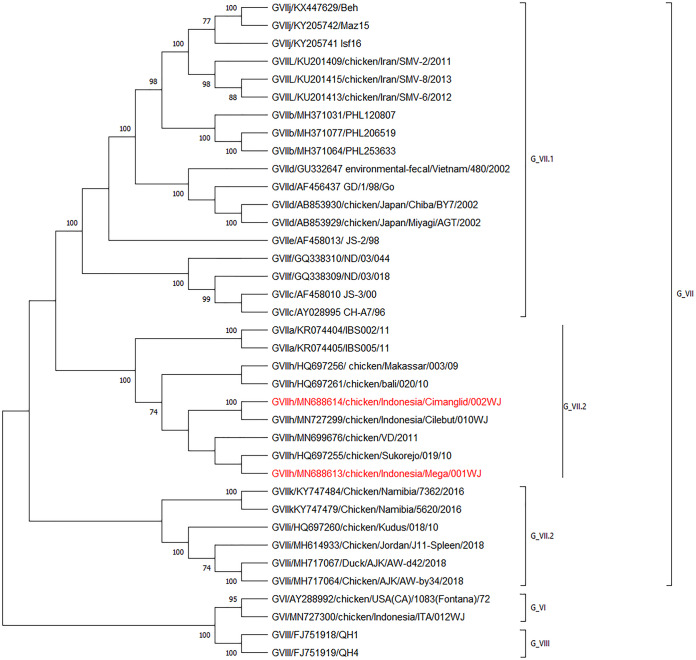
Phylogenetic analysis based on the full-length fusion protein gene of representative NDV isolates. The evolutionary history was inferred by using the maximum likelihood method and general time-reversible model in MEGA X. The tree with the highest log likelihood (−7,196.50) is shown. The percentage of trees in which the associated taxa clustered together is shown next to the branches. Initial trees for the heuristic search were obtained automatically by applying neighbor-joining and BioNJ algorithms to a matrix of pairwise distances estimated using the maximum composite likelihood (MCL) approach and then selecting the topology with a superior log likelihood value. A discrete gamma distribution was used to model evolutionary rate differences among sites (five categories [+G; parameter, 1.0951]). The rate variation model allowed for some sites to be evolutionarily invariable (+I; 33.02% of sites). This analysis involved 37 nucleotide sequences. Codon positions included were first, second, third, and noncoding. All positions with <95% site coverage were eliminated, i.e., fewer than 5% alignment gaps, missing data, and ambiguous bases were allowed at any position (partial deletion option). There were a total of 1,647 positions in the final data set. The vertical lines show genotype VII, with subgenotypes VII.1 and VII.2, genotype VI, and genotype VIII. The two virulent strains of subgenotype VII.2 in this study are highlighted in red.

Sequence analysis revealed that the two strains differed in the amino acid sequence at the C terminus of the F protein cleavage site, which is a key determinant of NDV pathogenicity (15, 16). The Cimanglid/002WJ strain encodes the amino acid sequence motif ^112^RRRKRF^117^, while the Mega/001WJ strain encodes ^112^RRQKRF^117^. Phylogenetic analysis carried out on F gene sequences using MEGA X (17) suggests that these circulating strains belong to NDV genotype VII.2 (Fig. 1), a cause of many recent disease outbreaks in Indonesia. The amino acid identities of NP, P, M, F, HN, and L proteins between the current virus strains and the LaSota vaccine strain that is most commonly used in Indonesia are 92%, 81%, 88%, 89%, 85%, and 94%, respectively. These differences could contribute to poor protection against these strains by NDV vaccines, which highlights the need for a vaccine development strategy against newly emerged NDV strains in Southeast Asia.

### Data availability.

The genome sequences for Mega/001WJ and Cimanglid/002WJ were deposited in GenBank with accession numbers MN688613 and MN688614, respectively. The raw sequence data were deposited in the NCBI Sequence Read Archive (SRA) under BioProject number PRJNA613298.
